# Internalized stigma: Social support, coping, psychological distress, and mental well-being among older adults in Ghana

**DOI:** 10.1177/00207640241227128

**Published:** 2024-02-07

**Authors:** Mabel Oti-Boadi, Johnny Andoh-Arthur, Kwamina Abekah-Carter, Daniel Naawenkangua Abukuri

**Affiliations:** 1 Department of Psychology, University of Ghana, Accra, Ghana; 2 School of Social Work, Memorial University of Newfoundland, St. John’s, Canada

**Keywords:** Internalized stigma, social support, coping, mental well-being, older adults

## Abstract

**Background:**

Older adults have been found to internalize stigma from society and this has been linked to several variables including social support, coping, psychological distress, and mental well-being. However, there is a dearth of research on how these variables interact with each other to impact the life of older adults.

**Aims:**

This study employed path analysis to explore social support and coping as boundary conditions and underlying mechanisms in the link between internalized stigma, psychological distress, and mental well-being, among older adults in Ghana.

**Method:**

Using a cross-sectional design, the study recruited 167 older adults who responded to standardized questionnaires including The Internalized Stigma of Mental Illness scale, Multidimensional Perceived Social Support Scale, The Brief Coping Inventory, Kessler Distress Scale, and the Warwick-Edinburgh Mental Well-being Scale, to determine levels of internalized stigma, social support, coping strategies, psychological distress, and mental well-being, respectively.

**Results:**

The findings revealed that at low levels of social support, there is a significant and positive correlation between internalized stigma and mental well-being (β = −.36, *SE* = 0.17, *p* *<* .001). Path analysis showed that the relationship between internalized stigma and mental well-being was fully mediated by problem-focused coping (β = .11, *p* = . 001, 95% CI [0.04, 0.21]), but not psychological distress. Problem-focused coping was also found to have a significant positive correlation with mental well-being but no significant correlation with psychological distress. Conversely, avoidant-focused coping was found to have a significant positive correlation with psychological distress and a significant negative correlation with mental well-being.

**Conclusions:**

This study revealed the importance of social support and coping to attaining mental well-being among older adults. This study provides insights into the development of tailored interventions aimed at improving social support and problem-focused coping among older Ghanaian adults facing internalized stigma, and it also establishes a base for future research.

## Introduction

There is a current global demographic shift characterized by a significant rise in the aging population (Nagarajan & Sixsmith, 2023). It is estimated that there are more than one billion older adults in the world (World Health Organization, 2022), and the number of older adults who reside in Ghana accounts for about 6.7% of the national population (Ghana Statistical Service, 2021). Nonetheless, this demographic transition is associated with some unique challenges, including older adults’ vulnerability to discrimination and stigma from family and society (Kang & Kim, 2022). Stigma is often associated with mental illness and the population of persons with mental illness (Mock & Eibach, 2011). However, there is evidence that other social groups, including older adults also suffer from stigma due to the ageing process (Bryant & Koder 2014; Werner et al., 2009).

Some studies have shown that the negative perceptions associated with old age have an undesirable impact on the memory and cognitive processing of older adults (Giasson & Chopik, 2020; Lamont et al., 2015). Additionally, these negative perceptions have been associated with increased stress reactivity among older adults (Levy et al., 2000; Weiss, 2018). In Ghana, the admiration for older adults as custodians of knowledge and culture is paradoxically accompanied by various forms of ageist stereotypes, including witchcraft accusations (Mabefam & Appau, 2020). Such stigma-related acts could lead to various issues, including suicidal ideation (Abekah-Carter & Oti, 2022; Adinkrah 2020).

Stigma-related stereotypes increase the emergence of internalized stigma among older adults (Sun et al., 2022) and serve as a threat to their well-being (Marquet et al., 2018; Tzouvara et al., 2018). Internalized stigma is the phenomenon in which individuals attribute detrimental stereotypes to themselves, anticipate rejection from others, and experience a sense of isolation from society (Livingston & Boyd, 2010). Internalized stigma has been associated with several negative outcomes, including increased anxiety, depression, low self-esteem (Emile et al., 2014; Wurm & Benyamini, 2014), limited involvement in social activities (Weiss & Lang, 2012), and reduced access to social support and health services (Levy, 2003). Well-being can be understood from two main perspectives namely, hedonic and eudaimonic (Ryan & Deci, 2001). Whilst hedonic concerns the attainment of pleasure and the avoidance of pain (Keyes et al., 2002; Ryff, 1989; Serie et al., 2021), eudaimonic consists of seeking self-realization and meaning (Extremera et al., 2011; Ryff, 1989).

Extant literature has found social support as a potential moderator in the relationship between internalized stigma and psychological distress and psychological well-being (Ajrouch et al., 2010; Cobo-Rendón et al., 2020; Park & Joshanloo, 2022). Low levels of social support have also been linked to higher levels of societal and internalized stigma, and lower levels of recovery and quality of life among adults with mental illness (Chronister et al., 2013). However, the actual mechanism through which internalized stigma affects psychological distress and well-being remains unclear.

Coping plays an important role in internalized stigma, well-being of different age groups (Mayordomo-Rodríguez et al., 2015). According to Phelan et al. (2004) coping is an important factor when it comes to successful aging. Coping refers to strategies that are adopted to overcome adversities based on available resources (Marquez-Arrico et al., 2015), and is also linked to social support and well-being (Folkman & Moskowitz, 2004; Schoenmakers et al., 2015). Studies have found a positive relationship between coping strategies, such as problem-coping, emotion-coping, and resilience and subjective well-being of older persons (Galiana et al., 2020; Tovel & Carmel, 2014). Whilst in the short-term, maladaptive coping strategies may help reduce symptoms of distress, in the long term, the consequences may be dire (Jacofsky et al., 2020). Research has shown coping strategies such as secrecy, withdrawal, emotional coping, and tangible support mediate the effect of societal stigma on internalized stigma and recovery (Chronister et al., 2013). Moreover, a significant indirect effect of HIV-related discrimination on cognitive escape coping through internalized stigma was found among some adults with HIV and mental illness (Guy et al., 2022). Studies have shown that among older adults, having positive attitudes toward their ageing process may cushion against mental illness and experience improved mental well-being (Kim, 2015; Levy & Myers, 2004).

### The present study

Despite these efforts at research and considering the links between internalized stigma psychological distress, psychological well-being, social support, and coping, to the knowledge of current researchers, no study has been conducted in Ghana among older adults to examine whether their internalized stigma contributes to their psychological well-being. For older adults in a country like Ghana where cultural norms play a crucial role in shaping perceptions about vulnerable populations (Doku, 2012), the impact of internalized stigma may be particularly pronounced. Therefore, this study aimed to enhance the understanding of internalized stigma among Ghanaian older adults and to further explore the relationships between experiences of internalized stigma and mental health variables, such as social support, coping, psychological distress, and mental well-being. Identifying and understanding the psychological processes associated with internalized stigma among older adults will significantly contribute to the development of tailor-made interventions being considered by policymakers on how to effectively manage and support older adults. The study hypothesizes that internalized stigma will significantly predict psychological distress and well-being. Secondly, coping strategies will fully mediate the relationship between internalized stigma, psychological distress, and mental well-being in expected directions. Thirdly social support will moderate the relationship between internalized stigma and mental well-being.

## Methods

### Participants

Using purposive and convenience sampling methods, the study recruited 167 participants aged 50 years and above from a support group for older adults and other urban communities in Accra, Ghana. With the support of research assistants, eligible older persons were assisted in answering the study questions. The sample size was determined following the method and standard requirements of the cross-sectional sample size outlined by Zhou et al. (2017).

Of the 167 participants were eligible for the study, there was a slightly higher representation of males (50.3%) compared to females (49.7%). Many of the participants identified as Christians (85.8%), and a substantial portion reported being married (65.9%). The mean age of participants was *M* = 60.86 years, with a standard deviation of *SD* = 10.48 years. The study’s inclusion criteria comprised older adults who were fluent in English (the Lingua Franca of Ghana), gave their consent, were physically and mentally fit, had no diagnosis of Dementia, had clear cognition, and had no serious hearing impairments. Participation in the study was voluntary, and informed consent was obtained from each participant. Participants failing to meet the eligibility criteria particularly included those outside the specified age range, and those not residing in the study setting. In this study, it was possible for some participants who met the inclusion criteria to be excluded due to issues, such as health decline during data collection, withdrawal of informed consent, and unforeseen circumstance. While none of these were observed in this study, the researchers ensured they were prepared to address such issues.

### Measures

#### Internalized stigma: The Internalized Stigma of Mental Illness (ISMI) scale

The ISMI scale is the most used instrument for the evaluation of internalized mental illness stigma (Ritsher et al., 2003). It has been adapted to some other populations or problems showing good psychometric properties in all cases. This scale was adapted to measure internalized stigma among older adults (González-Dominguez et al., 2018). ‘For example, Item 1, which states: *I feel out of place in the world because I feel out of place in the world because I am old have a mental illness,* was changed to, *I feel out of place in the world because I am old*’. The ISMI is a 29-item instrument in which participants rate their degree of agreement with the statement expressed on a 4-point Likert scale ranging from (1 = ‘strongly disagree’ to 4 = ‘strongly agree’). The items are grouped into five factors: alienation (feeling inferior to other members of society); stereotype endorsement (the degree to which one agrees with the most common stereotypes); discrimination experience (the interviewee’s perception of the way that others treat him/her); social withdrawal (an important factor of exclusion); and stigma resistance (the experience of refusing to be affected by the stigma; Ritsher et al., 2003). Items 22 to 26 reverse coded. A high total score on the ISMI scale indicates more severe internalized stigmatization. The overall Cronbach alpha for this scale is .89 (González-Dominguez et al., 2018).

#### Coping: The Brief Coping (BC) Inventory

The Brief Cope Inventory is a 28-item questionnaire developed from the original version of the Cope Inventory which was made up of 60 items (Carver, 1997). Thus, this inventory offers a faster and more convenient way of assessing coping strategies. This scale assesses 14 different coping reactions (factors) which could be generally adaptive or maladaptive strategies. The 28 items on the Brief Cope Inventory measure 14 factors, covered by two items each. They include Self-distraction (1 and 19); Active coping (2 and 7); Denial (3 and 8); Substance use (4 and 11); Use of emotional support (5 and 15); Use of instrumental support (10 and 23); Behavioral disengagement (6 and 16); Venting (9 and 21); Positive reframing (12 and 17); Planning (14 and 25); Humor (18 and 28); Acceptance (20 and 24); Religion (22 and 27); and Self-blame (13 and 26). The response format ranges from 1 (‘I haven’t been doing this at all’) to 4 (‘I’ve been doing this a lot’). Higher scores reflect better coping strategies while lower scores mean poor coping strategies, with the total highest score of 112 and the lowest possible score being 28. García et al. (2018) reported internal consistencies ranging from α = .53 to .82.

#### Social support: Multidimensional Scale of Perceived Social Support (MSPSS)

The 12-item Multidimensional Scale of Perceived Social Support (MSPSS) measures a person’s perception of their amount of social support. It comprises three subscales that assess the social support received from friends, family, and significant others. Each of the three subscales has four items. The response scale ranging from 1 (*very strongly disagree*) to 7 (*very strongly agree*) was used for measurement. According to Zimet et al. (1988), the Significant Other, Family, and Friends subscales had test-retest reliability of .72, .85, and .75, respectively. The reliability index achieved for the entire scale is.85.

#### Mental Well-Being Scale

The Warwick-Edinburgh Mental Well-Being Scale (WEMWBS) was used to measure positive mental health (Tennant et al., 2007), that is, the degree to which participants were thriving or flourishing in their lives. The WEMWBS covers the two eudaimonic and hedonic aspects of positive mental health and appears to have good reliability and validity as a measure of positive mental health (Stewart-Brown et al., 2009; Tennant et al., 2007). It consists of 14 items, and participants are asked to respond according to their experience over the past 2 weeks on a 5-point scale ranging from 1 to 5. Item scores are added to produce a total score between 14 and 70, with higher scores indicating greater positive mental health. Cronbach alpha for this scale is .87 (Clarke, 2011).

#### Psychological distress

The Kessler Psychological Distress Scale (K10) is a measure of psychological distress. The K10 scale involves 10 questions about emotional states each with a five-level response scale. The measure can be used as a brief screen to identify levels of distress. The tool can be given to patients to complete, or the questions can be read to the patient by the practitioner. Each item is scored from one *(none)* to five *(all of the time)*. A factor analysis using a varimax rotation method revealed that all 10 items measure the same underlying construct (Factor loadings ranging from 0.51 to 0.81, with 51% variance explained). Hence, the scores of the 10 items were summed to form the Psychological Distress scale, with an alpha value of .89, suggesting very high reliability. Lower scores indicate low levels of psychological distress, and high scores indicate high levels of psychological distress.

### Procedure

Ethics approval was obtained from the Departmental Research and Ethics Committee, Department of Psychology, University of Ghana (DREC/009/22-23). The ethical approval letter and the purpose of the study were shared with a support group for older adults in Accra. The lead investigator works for the support group and thus used her internal contacts to facilitate the permission. When permission was granted, the lead Investigator worked closely with the authorities of the support group to recruit older people who met the eligibility criteria of the study. For those older adults who voluntarily consented to participate in the research, a set of measures assessing internalized stigma, coping strategies, social support, psychological distress, well-being as well as demographic questions was given to them to complete. Those who could not read were assisted by a translator to complete the questionnaires. The survey lasted for 30 minutes and was then returned. While the study sought to work within this time frame, flexibility was also introduced to have the measures filled at a place and time of convenience to the participants.

## Results

Table 1 shows the descriptive statistics, bivariate correlation, and reliability coefficient of the study. Assessment of internal consistency shows that all the main variables have alpha values above the acceptable threshold (*⩾*.70). Therefore, all the main variables: internalized stigma (α = .94), social support (α = .95), problem-focused coping (α = .88), emotion-focused coping (α = .84), avoidant-focused coping (α = .79), psychological distress (α = .94), and well-being (α = .95) are appropriate for statistical analysis in the present study. Internalized stigma correlated significantly to problem focused coping, *r* = .22, *p* = .004, emotion focused coping, *r* = .41, *p* *<* .001, avoidant coping, *r* = .57, *p* *<* .001, psychological distress, *r* = .71, *p* *<* .001, and well-being, *r* = −.29, *p* *<* .001, but insignificantly to social support, *r* = −.04, *p* = .598. The results also indicated that social support correlated significantly to well-being, *r* = .26, *p* *<* .001, but insignificantly to problem-focused, *r* = .05, *p* = .499, emotion-focused, *r* = −.01, *p* = .849, avoidant focused, *r* = −.06, *p* = .461, and psychological distress, *r* = −.03, *p* = .655. Furthermore, it was observed that problem-focused coping correlated significantly with emotional-focused coping, *r* = .79, *p* *<* .001, avoidant coping, *r* = .45, *p* *<* .001, and psychological distress, *r* = .20, *p* = .008, but insignificantly to well-being, *r* = .13, *p* = .091. It was also revealed that emotion-focused coping correlated significantly with avoidant coping, *r* = .65, *p* *<* .001, psychological distress, *r* = .39, *p* *<* .001, but insignificantly to well-being, *r* = −.04, *p* = .642. Avoidant coping related significantly to psychological distress, *r* = .64, *p* *<* .001, and well-being, *r* = −.27, *p* *<* .001. Finally, psychological distress related significantly to well-being, *r* = −.39, *p* *<* .001.

**Table 1.** Descriptive statistics, bivariate correlation, and reliability coefficient results of the study variables.

No.	Variables	*M*	*SD*	1	2	3	4	5	6	7	8	9	10	11	12	13
1.	Gender	.50	.50	−												
2.	Age	60.86	10.04	−0.04	–											
3.	Religion	0.17	0.45	0.02	0.14	–										
4.	Marital status	1.60	1.22	−0.15*	0.52***	0.17*	–									
5.	Education	3.72	1.35	0.21**	0.37***	−0.12	−0.34***	–								
6.	Employment status	1.48	0.94	−0.15	0.16*	0.11	0.09	−0.44***	–							
7.	Internalized stigma	2.05	0.52	−0.02	0.34***	0.10	0.27***	−0.30***	0.22**	(0.94)						
8.	Social support	4.75	1.54	−0.02	0.01	−0.00	0.00	0.17*	−0.06	−0.04	(0.95)					
9.	Problem-focused	2.66	0.80	−0.11	−0.06	0.04	−0.00	−0.01	0.08	0.22**	0.05	(0.88)				
10.	Emotion-focused	2.39	0.64	−0.07	0.07	0.08	0.11	−0.14	0.12	0.41***	−0.01	0.79***	(0.84)			
11.	Avoidant focused	2.01	0.64	0.10	0.23**	0.14	0.20	−0.19*	0.18*	0.57***	−0.06	0.45***	0.65***	(0.79)		
12.	Psychological distress	2.29	0.98	−0.06	0.39***	0.16	0.28***	0.21**	0.26***	0.71***	−0.03	0.20**	0.39***	0.64***	(0.94)	
13.	Well-being	3.63	0.90	0.04	−0.16*	−0.16	−0.30***	−0.10	−0.29***	−0.29***	0.26***	0.13	−0.04	−0.27***	−0.39***	(0.95)

*Note*. Reliability coefficients in parenthesis.

* *p* < .05. **p < .01. ***p < .001.

### Testing hypotheses

We employed a hierarchical multiple regression test to assess the moderation hypotheses. However, the mediation hypotheses were tested using path analysis because path analysis allows for the testing of specific indirect effects (i.e. mediation effects) when more than one mediator is involved. Consequently, the present study utilized the SPSS version 26 and SPSS AMOS version 26 for IBM to facilitate the testing of the moderation and mediation hypotheses, respectively.

### Moderation analysis

The hierarchical multiple regression analysis results showed that the effect of social support and internalized stigma on well-being is consistent across the models: Model 2 (direct effect) and Model 3 (interactive effect). Therefore, the results in Model 3, which contain the direct and interactive effects were interpreted. Specifically, the result shows that social support significantly and positively predicted well-being, β = .28, *SE* = .04, *p* *<* .001, highlighting that increased levels of social support were associated with improved well-being. Therefore, the third hypothesis is supported. Also, the study showed that internalized stigma significantly and negatively predicted well-being, β = −.37, *SE* = .13, *p* = .004, supporting hypothesis 1. This outcome implies that high levels of internalized stigma were associated with low well-being.

**Figure 1.**
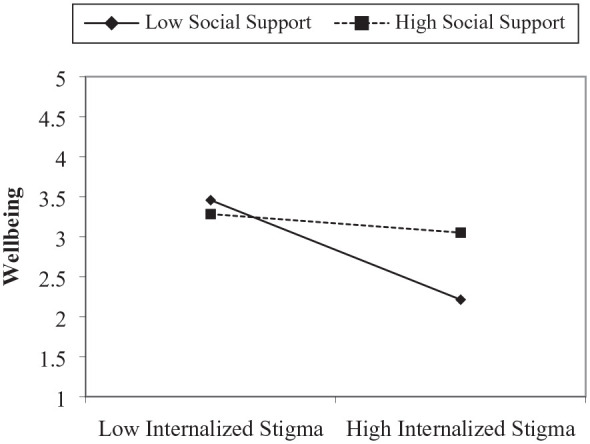
Slope analysis.

Before testing the moderation hypothesis, the researcher centered the independent (i.e. internalized stigma) and moderator (i.e. social support) variables. The centered independent and moderator variable was then multiplied to create the interactive term (i.e. internalized stigma x social support). The result showed that social support significantly moderated the relationship between internalized stigma and well-being, β = .23, *SE* = .07, *p* = .001. To determine the level of the moderator at which internalized stigma predicts well-being, we followed the recommendation by Aiken and West (1991) to perform slope analysis at the +/1 *SD* below the mean. Figure 1 shows the slope analysis, which indicates that internalized stigma significantly and negatively predicted well-being at a low level of social support, β = −.36, *SE* = .17, *p* *<* .001, but the relationship was insignificant at a high level of social support, β = −.07, *SE* = .135, *p* = .390 (Table 2).

**Table 2.** Hierarchical multiple regression results of the effect of social support and internalized stigma on well-being.

	Well-being					
	Model 1		Model 2		Model 3	
	β	*SE*	β	*SE*	β	*SE*
Constant	3.96	.55	3.97	.54	3.98	.52
Age	.00 (.02)	.01	.00 (.05)	.01	.00 (.05)	.01
Religion	−.17 (−.08)	.15	−.16 (−.08)	.14	−.14 (−.07)	.13
Marital status	−.19 (−.25)**	.06	−.19 (−.25)**	.06	−.19 (−.25)**	.06
Educational level	.05 (.08)	.06	.00 (.01)	.06	−.01 (−.01)	.05
Employment status	−.22 (−.23)**	.08	−.21 (−.21)**	.07	−.18 (−.18)**	.07
Social support	–	–	.14 (.24)**	.04	.17 (.28)***	.04
Internalized stigma	–	–	−.30 (−.17)*	.13	−.37 (−.21)**	.13
Social support x internalized stigma	–	–	–	–	.25 (.23)**	.07
*R* ^2^	.17***		.26***		.31**	
*∆R* ^2^	.17***		.08***		.05**	
*F*	7.08***		8.12***		9.07***	

*Note*. Standardized beta values are in parenthesis.

* *p* < .05. **p < .01. ***p < .001.

### Mediation analysis

The study employed path analysis to test the extent to which internalized stigma predicted well-being and psychological distress via problem-focused and avoidant-focused coping, respectively. In testing for the mediation effect, we assessed direct and indirect effects simultaneously. Results of the direct effect analysis indicated that internalized stigma, β = .22, *p* = .003, 95% CI [0.07, 0.34] significantly and positively related to problem-focused coping; avoidant coping, β = .57, *p* = .001, 95% CI [0.42, 0.68]; and psychological distress, β = .50, *p* = .001, 95% CI [0.33, 0.65], however, internalized stigma related insignificantly to well-being, β = −.18, *p* = .140, 95% CI [−0.39, 0.06]. Furthermore, problem-focused coping related significantly and positively to well-being, β = .30, *p* = . 001, 95% CI [0.17, 0.43], but insignificantly to psychological distress, β = −.09, *p* = .080, 95% CI [−0.19, 0.01]. Finally, it was found that avoidant-focused coping related significantly and positively to psychological distress, β = .40, *p* *<* .001, 95% CI [0.23, 0.56], and negatively and significantly to well-being, β = −.31, *p* = . 004, 95% CI [−0.52, −0.09]. The study employed the bootstrapping approach to testing the indirect effect of problem and avoidant-focused coping on the link between internalized stigma, well-being, and psychological distress. We used 5,000 bootstrap bias-corrected samples to test for the indirect effect. The results showed that internalized stigma significantly affected well-being via problem-focused coping, β = .11, *p* = .001, 95% CI [0.04, 0.21], but problem-focused coping insignificantly mediated the relationship between internalized stigma and psychological distress, β = −.04, *p* = .053, 95% CI [−0.10, 0.00]. Finally, the results indicated that internalized stigma significantly affected well-being, β = −.30, *p* = .003, 95% CI [−0.54, −0.09] and psychological distress, β = .43, *p* *<* .001, 95% CI [0.26, 0.64] (Table 3).

**Table 3.** Path analysis results of well-being and psychological distress.

Models		95% confidence interval	
Direct effects	Estimates	LL	UL
Internalized stigma → problem-focused coping	.22**	0.07	0.34
Internalized stigma → avoidant-focused coping	.57**	0.42	0.68
Internalized stigma → well-being	−.18	−0.39	0.06
Internalized stigma → psychological distress	.50**	0.33	0.65
Problem focused → well-being	.30**	0.17	0.43
Problem focused → psychological distress	−.09	−0.19	0.01
Avoidant focused coping → psychological distress	.40***	0.23	0.56
Avoidant focused coping → well-being	−.31**	−0.52	−0.01
Indirect effects			
Internalized stigma affects well-being via problem-focused coping	.11**	0.04	0.21
Internalized stigma affects psychological distress via problem-focused coping	−.04	−0.10	0.00
Internalized stigma affects well-being through avoidant focused coping	−.30**	−0.54	−0.09
Internalized stigma affects psychological distress via avoidant-focused coping	.43***	0.26	0.64

** *p* < .01. ***p < .001.

## Discussion

The objective of the current study was to examine the relationships between internalized stigma, social support, coping, and mental well-being among older adults in Ghana. Increased internalized stigma was found to be associated with lower mental well-being among the older adults, aligning with existing literature. (Bryant et al., 2012; Mock & Eibach, 2011). Negative stereotypes towards older persons can make them susceptible to mental health issues including depression, anxiety, and suicide ideation (Marquet et al., 2019). In this study, internalized stigma also correlated significantly with all coping variables (e.g. emotion-focused, problem-focused, and avoidant-focused coping), but not with social support. In reference to the study’s hypotheses, whilst internalized stigma significantly and negatively predicted well-being, social support significantly and positively predicted well-being, indicating that increased levels of social support were associated with improved well-being. Moreover, it was subsequently found that social support moderated the relationship between internalized stigma and well-being. This finding is consistent with studies, such as Li et al.’s (2020) that indicated that social support could play a pivotal moderating role between internalized stigma and issues associated with one’s well-being, including mental illness.

Further, the study found that internalized stigma significantly predicted well-being at low levels of social support, but increased social support did not affect the relationship. These findings provide a strong indication of the role of limited social support for older adults in how they internalize stigma. This corroborates existing findings where low levels of social support have been linked to higher levels of societal and internalized stigma and lower levels of recovery and quality of life among adults (Chronister et al., 2013).

Generally, the current findings resonate with the literature where social support serves as a moderator in the relationship between internalized stigma and psychological distress and psychological well-being (Ajrouch et al., 2010; Cobo-Rendón et al., 2020; Park & Joshanloo, 2022). However, having internalized stigma has been blamed for older persons hesitating to seek support and thus accounting for the nonsignificant relationship between internalized stigma and well-being at higher levels of social support (Al-Dwaikat et al., 2022; Goreis et al., 2020). Notably, internalized stigma predicted well-being in the current study. This finding shows how strong internalized stigma could affect the well-being of older adults in Ghana. This could be explained through the transactional process where internalized stigma may prevent older adults from sharing their problems, which further results in limited social interaction and social support (Al-Dwaikat et al., 2022). It is well noted that improving social support systems amounts to using a problem-focused coping that contributes to well-being among older persons and alleviating the stress associated with stigma from society (Dumitrache et al., 2019; Schoenmakers et al., 2015). The relationships among the various variables under study present an indication that supports the development of tailor-made interventions to address ageism, internalized stigma, psychological distress, well-being, and social support.

In the path analysis, internalized stigma significantly predicted psychological distress and well-being. Internalized stigma also predicted problem-focused coping, avoidant coping, psychological distress, and well-being significantly and positively. Avoidant-focused coping related significantly and positively to psychological distress, and negatively and significantly to well-being. According to Goffman (1963), internalizing the negative stereotypes from society can negatively influence one’s well-being and may lead to the adoption of avoidant coping strategies to cope with stigma and psychological distress. Guy et al. (2022) found that among some adults living with HIV and mental illness, there was a significant indirect effect of HIV-related discrimination on cognitive escape coping through internalized stigma. Overall, avoidant coping strategies may prevent older adults from seeking help (Rotenberg et al., 2020).

Moreover, problem-focused coping significantly predicted psychological distress and well-being. This is in line with recent findings by Galiana et al. (2020), who found in their study that problem-focused coping significantly predicted both subjective and psychological well-being among older persons. Problem-focused coping mediated the relationship between internalized coping and well-being, but not psychological distress. This corroborates the finding that problem-focused coping supports older adults in adapting to stressful situations, thereby enhancing their well-being (Reyes et al., 2021). Thus, the current findings emphasize that understanding how internalized stigma develops, and how it interacts with other psychological variables is important for identifying and developing interventions to reduce internalized stigma. Despite this, research has shown that older adults are more likely to use emotion-focused coping than problem-focused coping (Chen et al., 2018). Older adults were also found to have adopted avoidant coping strategies, which related positively with psychological distress and negatively with well-being. Some studies have shown that the use of avoidant coping among older people could lead to physical health problems like higher levels of cortisol, problem drinking (Moos et al., 2006; O’Donnell et al., 2008), and psychological distress in the form of anxiety and suicidal ideation (Ayers et al., 2010; Woodhead et al., 2014). With regard to interpersonal tensions such as ageism, older adults may use avoidance coping as a strategy to steer clear of these interpersonal tensions (Huo et al., 2021). However, this has been found to be a risk to the sustenance of social relationships (Birditt et al., 2009). Therefore, recognizng the importance of coping strategies in the lives of older adults, it is essential to provide them with substantial support, particulary in developing problem-focused coping strategies. These strategies can assist them in effectively dealing with the emotional consequences associated with negative experiences in society (Etxeberria et al., 2018; Galiana et al., 2020; Schoenmakers et al., 2015).

## Implications to research, policy, and practice

From the research perspective, it would be interesting to examine older adults’ experience of internalized stigma in the Ghanaian context, as well as the protective resources available to them for coping. Qualitative studies could be done to gain deeper insights into the experiences of ageism in the Ghanaian community, and how and why older adults internalize it. Findings from such studies could inform the development of evidence-based and culturally sensitive strategies to address this issue in an efficient manner. Furthermore, the enactment of policies that support community-based initiatives that encourage social interactions among older adults could be useful in countering social isolation, which can be worsened by internalized stigma. In terms of practice, it is imperative for clinicians, social workers, and other helping professionals that work with older adults to take cognizance of their client’s experiences with internalized stigma and provide education on the negative impact on their mental well-being.

## Strengths and limitations of the study

Despite the novelty of this study in providing an understanding of the existing relationships between internalized stigma, social support, coping, psychological distress, and mental well-being, the study is also beset with some limitations. First, the convenience sampling technique used for the data collection could predispose our data to response bias where older adults with health problems may have been inadvertently included. Moreover, convenience sampling of older adults from a social group in Accra limits the generalization of the findings to other older adults in Ghana. Also, being in a social group itself could be a confounding factor. Second, the cross-sectional nature of the study prevents us from drawing causality conclusions. The current causal relationships are based on hypothetical relationships. It would be prudent to engage in longitudinal studies to examine causal relationships among the variables.

## Conclusion

This study highlights the relationship between internalized stigma, social support, coping strategies, psychological distress, and the mental well-being of older adults in Ghana. Higher internalized stigma was associated with poorer mental well-being, and with the various coping methods, but not with social support. Notably, social support moderated the relationship between internalized stigma and mental well-being especially at low levels of social support. However, path analysis demonstrated that internalized stigma significantly predicted psychological distress and mental well-being. Internalized stigma also positively predicted problem-focused coping, avoidant-focused coping, psychological distress, and mental well-being. Problem-focused coping also mediated the relationship between internalized stigma and mental well-being. The current study offers insights for tailored interventions aiding older Ghanaians adults facing internalized stigma, and it establishes a foundation for further research.
